# Deep learning enabled rapid detection of live bacteria in the presence of food debris

**DOI:** 10.1038/s41538-025-00636-z

**Published:** 2025-11-21

**Authors:** Hyeon Woo Park, Zhengao Li, Luyao Ma, Nitin Nitin

**Affiliations:** 1 Department of Food & Biotechnology, Korea University, Sejong, Republic of Korea; 2 Department of Food Science & Technology, University of California-Davis, Davis, CA USA; 3 Digital Healthcare Center, Sejong Institute for Business and Technology, Korea University, Sejong, Republic of Korea; 4 Department of Computer Science, Florida State University, Tallahassee, FL USA; 5 Department of Food Science & Technology, Oregon State University, Corvallis, OR USA; 6 Department of Biological & Ecological Engineering, Oregon State University, Corvallis, OR USA; 7 Department of Biological & Agricultural Engineering, University of California-Davis, Davis, CA USA

**Keywords:** Biotechnology, Microbiology

## Abstract

The contamination of food with pathogenic bacteria is a major public health concern, requiring rapid and accurate detection methods. Conventional approaches, such as culture-based or molecular assays, are time-consuming, labor-intensive, and often demand specialized expertise. Here, we developed a deep learning-based strategy for rapid detection and classification of live bacteria using simple white-light microscopic images of microcolonies, even in the presence of morphologically similar food debris. The model, based on ResNet50 with a Region Proposal Network, was trained on *Escherichia coli*, *Listeria monocytogenes*, *Bacillus subtilis*, and debris from chicken, spinach, and cheese. The model trained on bacteria misclassified debris as bacteria (24.2% false positives), whereas the model trained on both bacteria and food debris achieved 0% false positives with 100% precision and 94.4% recall. Validation with GFP-producing *B. subtilis* in food matrices further confirmed robust performance (mPrecision 94.6%, mRecall 92.5%). This cost-effective method enables reliable bacterial detection in complex foods within 3 h.

## Introduction

Bacterial contamination of food can occur throughout the entire production process, from pre-harvest on farms to post-harvest handling, processing, and the finished product. Bacterial contaminants can be introduced from multiple sources such as animals, irrigation water, soil, manufacturing facilities, and air^1–4^. Thus, early detection of foodborne pathogens before products are released to the market is essential to prevent outbreaks, protect consumer health, and minimize economic losses associated with food recalls and liability^5,6^. However, current bacterial detection methods either rely on standard cultivation techniques that take several days or require advanced instruments and specialized personnel, making them less accessible for the food industry^7^.

To address these challenges, deep learning-based approaches have emerged as promising tools for the rapid detection and classification of bacteria. Recent studies have demonstrated the potential of AI in this field. Kim et al. employed a support vector machine to classify five bacterial species on microfluidic chips, using bacteria and peptide-conjugated polystyrene suspensions^8^. Chen et al. developed a convolutional neural network to classify six common foodborne pathogens from microscopic images of gram-stained slides^9^. Yi et al. utilized a phage-induced bacterial lysis method to accurately detect viable *Escherichia coli* in water samples^10^. By using a convolutional neural network, the authors were able to distinguish between fluorescence images of lysed *E. coli* cells and intact cells of other bacterial species. In our previous study, we developed a deep learning-based method to detect and classify bacteria within 3 h using microscopic images of bacterial microcolonies grown on non-selective agar^5^. Although these studies highlight the potential of deep learning techniques for bacterial detection and classification, the impact of interference from various types of foods on the detection of foodborne bacteria using AI approaches has not yet been explored. Since most bacterial detection methods rely on imaging and spectral approaches, these interferences may include spectral overlap with food components, light scattering, background noise, variable contrast caused by food particles, and morphological similarities between bacteria and food debris^11–13^. These factors can obscure the detection and classification of bacterial targets and cause false positives, reduced classification accuracy, and difficulties in distinguishing bacteria from non-bacterial particles. Therefore, developing a robust deep learning-based model that can detect and classify foodborne bacteria in the presence of different food types is essential to enable the deployment of these approaches in the food industry.

This study aimed to evaluate the impacts of foodborne interferences on AI-enabled detection of bacteria from food samples using microscopy data and to develop deep learning-based solutions to minimize the interference of various food matrices in bacterial detection. Three types of food, including spinach, cheese, and chicken, were tested to represent fresh produce, dairy products, and meat. These food categories are widely consumed and associated with multiple foodborne recalls and outbreaks^14–17^. Mechanical disruption of these selected foods to prepare samples for bacterial detection and enumeration may generate diverse forms of microscopic particles or debris that can interfere with the microscopy-enabled AI-based detection of target bacteria. The selection of *E. coli*, *Listeria monocytogenes*, and *Bacillus subtilis* was based on their relevance to foodborne contamination and the distinct morphological characteristics of their microcolonies on agar media^18,19^. Furthermore, the structural similarity of these microcolonies’ features with diverse forms of food debris generated by common microbial sample preparation methods may influence the detection accuracy of AI-based approaches. White light microscopic images of bacterial microcolonies and food debris were obtained after 3-h incubation on standard agar plates. This incubation time was selected based on the results of our previous AI-enabled bacterial detection using microcolonies^5^. These image datasets were used to develop a robust deep learning-based bacterial detection model capable of discriminating bacterial microcolonies from food debris. This approach enhances the robustness of AI-enabled detection of target bacteria from realistic food samples, ultimately improving the reliability and applicability of AI models for food safety monitoring in the food industry.

## Results

### Comparative morphology of bacterial microcolonies and food debris

**Fig. 1:**
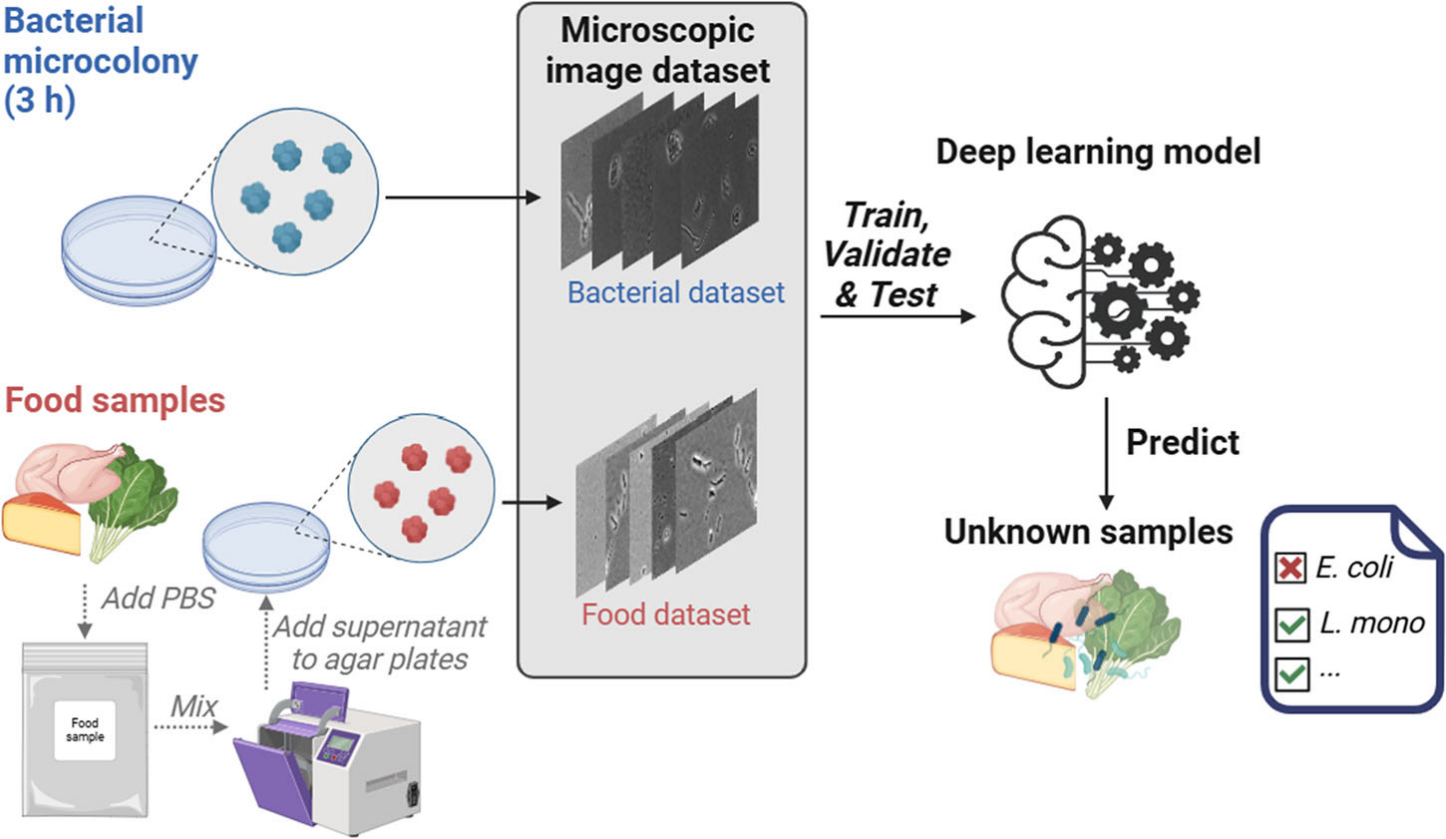
Pipeline of the AI-based bacterial detection model, illustrating the process from sample preparation to model application for the detection of target bacteria.

**Fig. 2:**
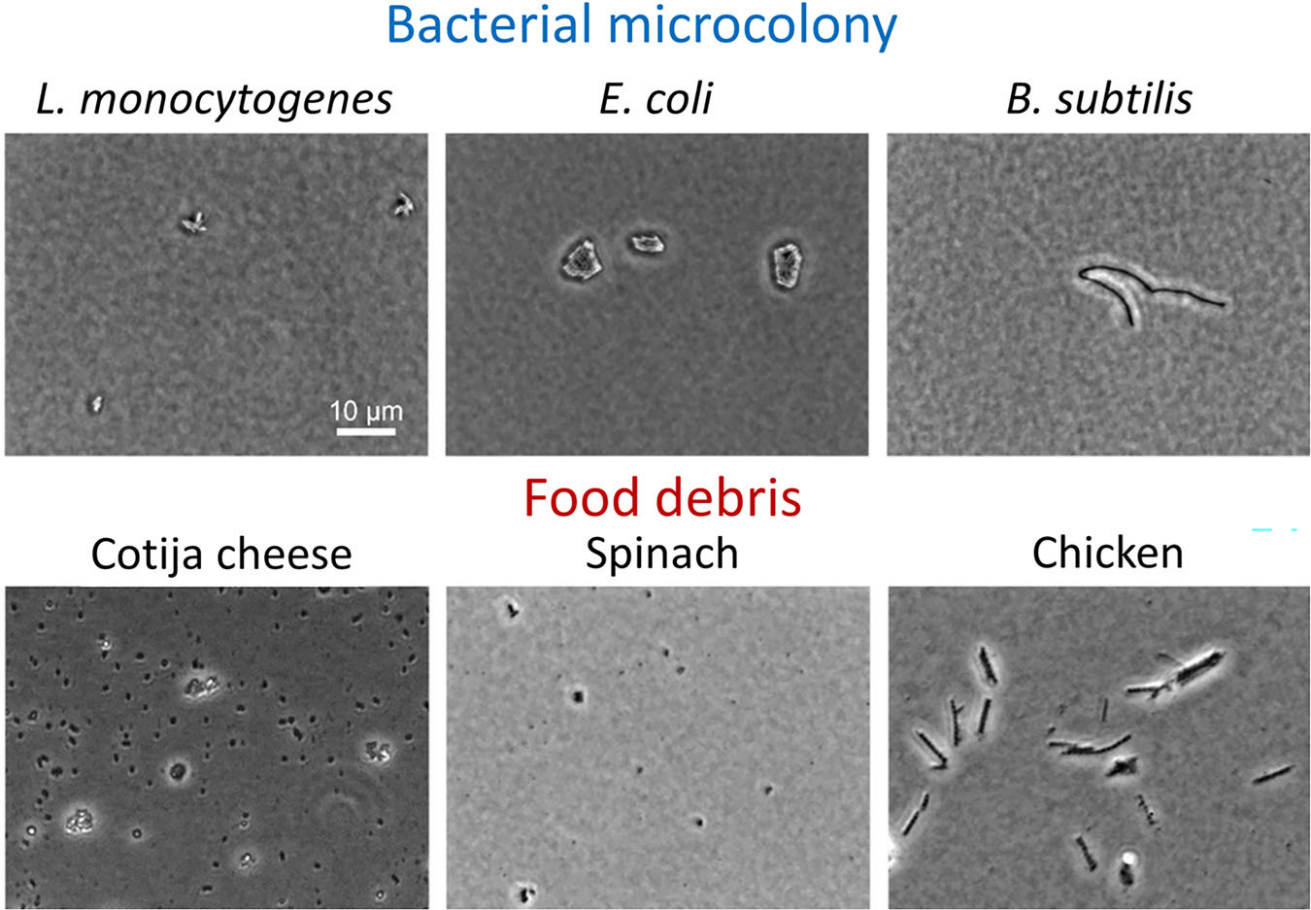
Representative images of bacterial microcolonies and food debris on agar plate captured using a phase-contrast microscope with a 60× objective.

The deep learning-based rapid bacterial detection approach developed in this study involves the following steps (Fig. 1): (1) incubation of bacteria samples using a standard bacterial plating method on TSA at 37 °C for 3 h, (2) preparation of food debris samples by homogenizing spinach, chicken breast, or Cotija cheese in PBS, followed by plating of the homogenized supernatant on TSA to mimic the standard food sample preparation workflow used for microbial analysis, (3) acquisition of microscopic images of bacterial microcolonies and food debris under simple white light illumination, (4) training the AI-based bacterial detection model with and without food debris images, and (5) application of the robust bacterial detection AI model in food matrices. The morphological characteristics of the target bacterial microcolonies alongside three representative food debris (chicken breast, spinach, and Cotija cheese) are shown in Fig. 2. The microcolonies of three bacterial species showed distinct morphological characteristics, which facilitate rapid bacterial classification within 3 h. *E. coli* formed rounded microcolonies, reflecting their rapid growth under the given incubation conditions. In contrast, *L. monocytogenes* produced microcolonies that were noticeably smaller than those of *E. coli*, which is attributed to the intrinsic slower growth rate of *L. monocytogenes*^20^. *B. subtilis* showed a rod-shaped microcolony, consistent with the characteristic rod shape of its individual cells^21^. Unlike the rounded macro-colonies of *B. subtilis* typically observed after culturing over a period of days^22^, the microcolonies observed after 3 h were more elongated or rod-like. This distinct morphology sets *B. subtilis* apart from both *E. coli* and *L. monocytogenes* microcolonies, which highlight the morphological diversity among these bacterial species at the early stage of growth (Fig. 2).

However, the morphologies of these bacterial microcolonies closely resembled selected food debris (Fig. 2) and may present a challenge for the accurate detection of bacterial microcolonies in the presence of food debris using manual visual determination. In the standard foodborne bacterial detection workflow, food samples are typically homogenized using a stomacher, resulting in various debris particles that can mimic bacterial microcolonies under a microscope. As shown in Fig. 2, debris from Cotija cheese and spinach appeared as small particles, similar in morphological appearance to the microcolonies of *L. monocytogenes* and *E. coli*. In contrast, the debris from chicken breast showed elongated, rod-shaped particles, resembling the microcolonies of *B. subtilis*. This resemblance is due to the fibrous nature of chicken breast^23^, which breaks into elongated fibers during homogenization. These morphological similarities highlight the critical need for the AI-based bacterial detection model to effectively distinguish bacterial microcolonies from food debris, as well as accurately classify the bacterial species. To address this, images of representative food debris were included in the pipeline of the rapid bacterial detection method development to enhance its practical application in the food industry.

### Deep convolutional neural network trained on bacterial images

**Fig. 3:**
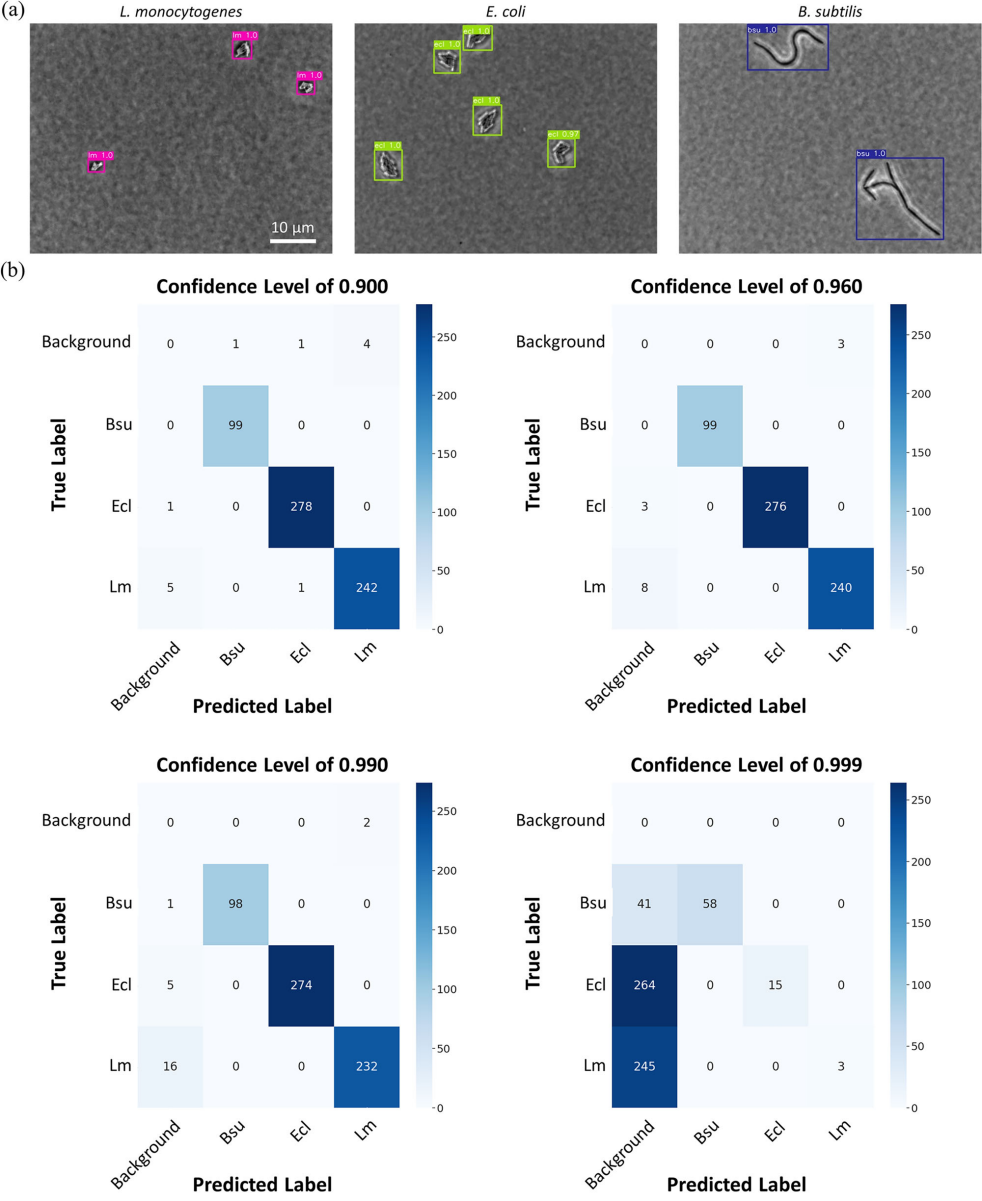
Bacterial classification using the deep convolutional neural network trained on *Listeria monocytogenes*, *Escherichia coli*, and *Bacillus subtilis.* **a** Representative images of object detection using the AI model to detect target bacteria; **b** Confusion matrices at different confidence levels.

To detect and classify bacteria, the architecture of the Faster R-CNN with two convolutional layers in the region proposal network (RPN) was employed^24^. To evaluate the model’s performance in classifying different bacterial species, images for each bacterial species were divided into three subsets: train (60%), validation (10%), and test datasets (30%), as described in the “Materials and Methods” section. During training, the validation dataset was used to optimize the hyperparameter settings. A separate test dataset was then used to evaluate the model’s performance on unseen data. As shown in Fig. 3a, the deep convolutional neural network model based on the ResNet50 backbone network and the RPN effectively detected and classified *L. monocytogenes*, *E. coli*, and *B. subtilis* due to their distinct morphological characteristics. To evaluate classification performance, confusion matrices were constructed at various confidence levels as shown in Fig. 3b. At a high confidence level of 0.999, the model showed 100% *mPrecision* due to the high threshold. However, many bacterial microcolonies were not detected, which caused *mRecall* to drop to 21.7%. At a confidence level of 0.990, the model maintained 99.7% *mPrecision*, while *mRecall* increased to 96.9% because the lower confidence level allowed for the detection of more microcolonies. At a confidence level of 0.960, the model achieved 99.6% *mPrecision* and 98.6% *mRecall*, which illustrates an improved balance between precision and recall. At a confidence level of 0.900, the model achieved a *mPrecision* of 98.9% and a *mRecall* of 99.1%, further improving detection sensitivity. These results indicate that setting an appropriate confidence level is necessary to achieve a balance between precision and recall.

**Fig. 4:**
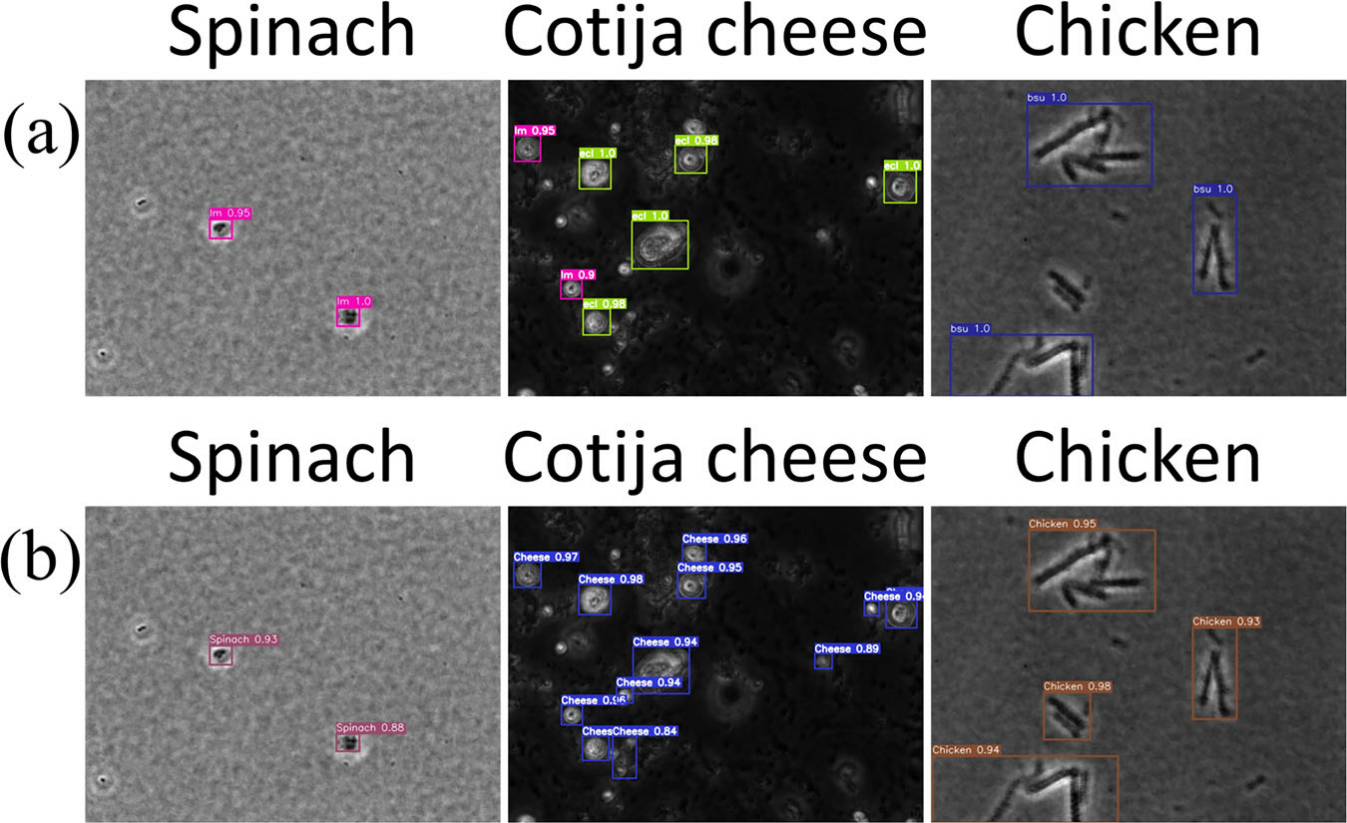
Object detection in food debris images using deep convolutional neural networks trained with and without debris. **a** Predictions based on the model trained using bacterial microcolonies; **b** Predictions based on the model trained using both bacteriaand food debris.

**Fig. 5:**
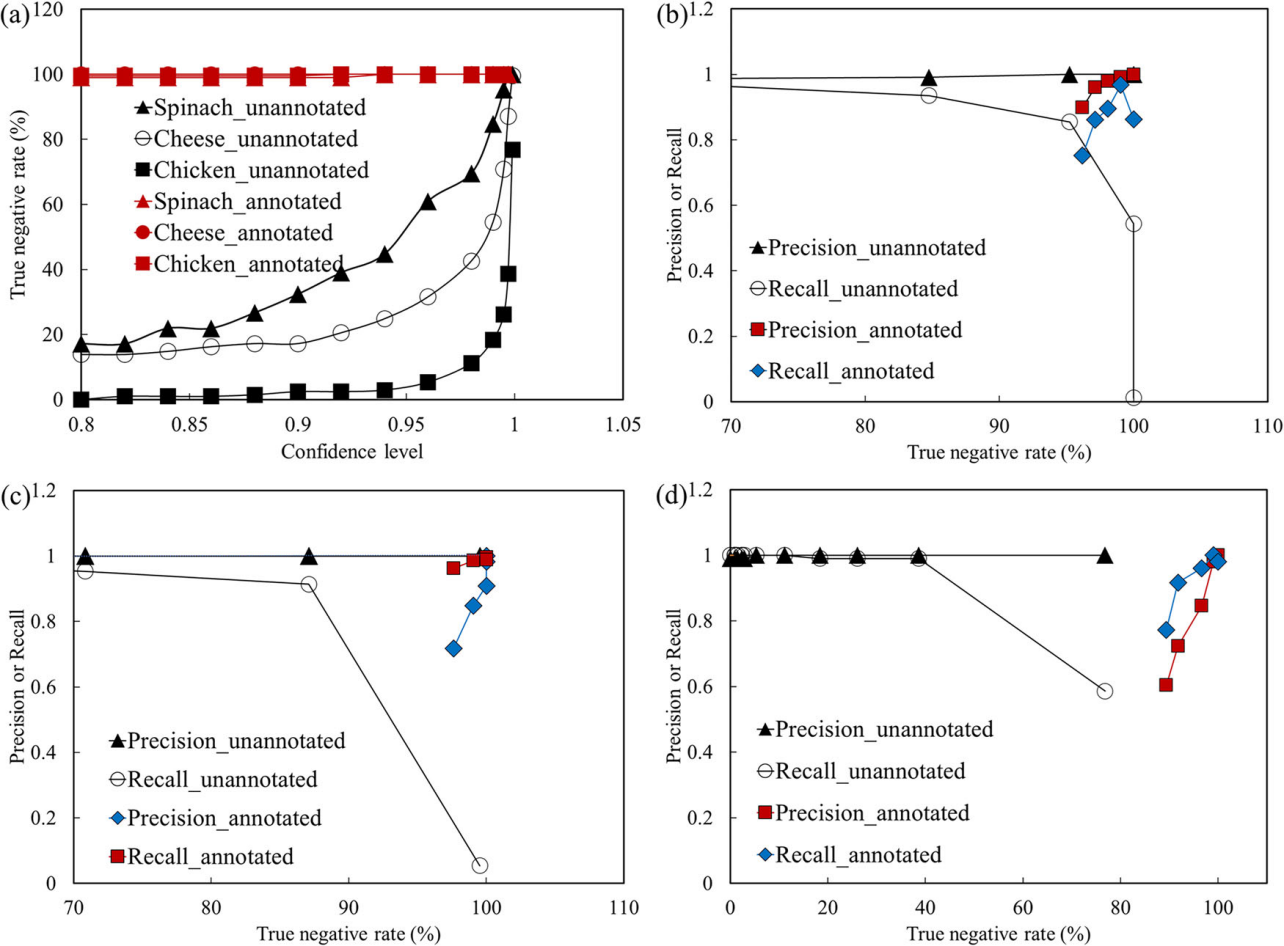
Influence of food debris on bacterial detection and classification performance. **a** True negative rates of food debris images at different confidence levels. Precision and recall curves of bacterial species in relation to the true negative rate of the corresponding food debris: **b** *Listeria monocytogenes* and spinach, **c** *Escherichia coli* and Cotija cheese, and **d** *Bacillus subtilis* and chicken breast. “Unannotated” and “annotated” refer to models trained on bacteria only and on bacteria with food debris, respectively.

To evaluate whether the bacterial classification model might misclassify food debris as bacteria, the model was further tested with the images of spinach, Cotija cheese, and chicken breast. Figure 4a showed that the model trained only on bacterial microcolonies reported the food debris as bacteria, indicating its limitations for real-life scenarios due to a high rate of false positives. Further testing with the food debris dataset was conducted to evaluate the true negative rate at different confidence levels (Fig. 5a). Chicken breast images showed the lowest true negative rates, which can be attributed to the morphological similarity between chicken breast fibers and the microcolonies of *B. subtilis*. All food debris had very low true negative rates (<20%) at a confidence level of 0.8, and the true negative rates gradually increased when the threshold of confidence levels increased. At a confidence level of 0.999, the model demonstrated high true negative rates for spinach and Cotija cheese samples, achieving rates of 100% and 99.5%, respectively (Fig. 5a). However, it is important to note that at this high confidence level, the model also failed to detect most of the bacterial microcolonies as discussed in the previous paragraph, resulting in *mPrecision* and *mRecall* of 100% and 21.7%, respectively.

### Improved deep convolutional neural network trained on bacteria and food

**Fig. 6:**
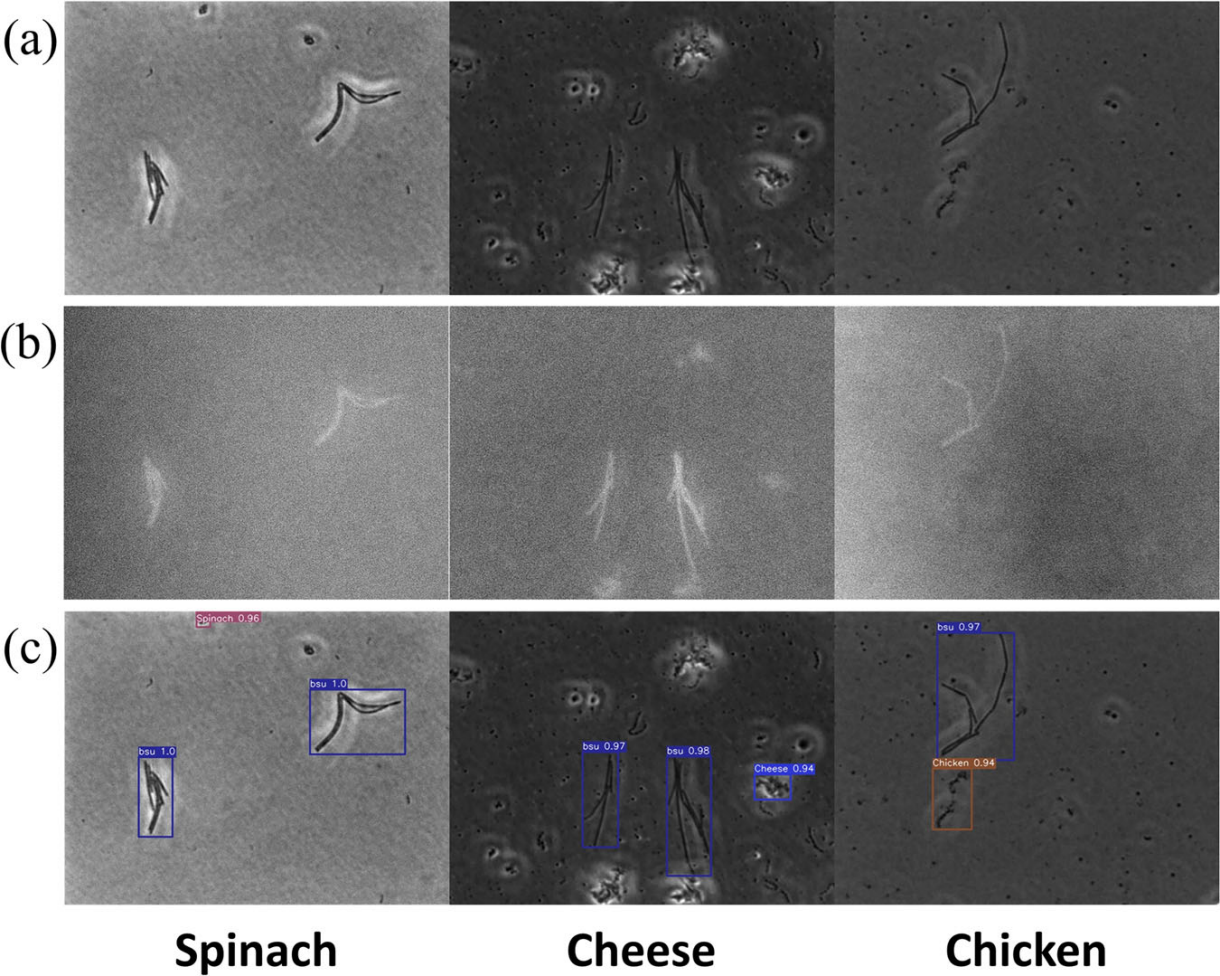
Validation of deep learning-based detection of *Bacillus subtilis* microcolonies in the presence of various food debris. **a** Phase-contrast image, **b** Fluorescence image, and **c** Model inference. Fluorescence images were used to locate *B. subtilis* microcolonies in the presence of food debris, serving as a reference for validating the deep learning-based bacterial detection.

To reduce the misclassification of food debris as bacteria while maintaining classification performance on bacteria, the deep convolutional neural network model was retrained by incorporating images of food debris collected from chicken breast, spinach, and Cotija cheese into the training dataset. This retrained model effectively distinguished food debris, which significantly reduced the false positive rate of bacterial detection (Fig. 4b). In this study, the retrained model is referred to as the “annotated” model (trained on both bacterial and food debris images), whereas the model trained solely on bacterial images is referred to as the “unannotated” model. The true negative rates for the food dataset at different confidence levels reflected this improvement, with spinach, Cotija cheese, and chicken breast achieving 100% true negative rates at confidence levels above 0.94 (Fig. 5a). Figure 6b-d illustrates the precision and recall curves of the improved model for *L. monocytogenes*, *E. coli*, and *B. subtilis* as functions of the true negative rate for spinach, Cotija cheese, and chicken breast, respectively. The results clearly showed that the deep convolutional neural network model trained on both bacterial microcolonies and food debris (annotated) maintained high classification performance (*Precision* and *Recall*) on bacterial species and high true negative rates for food samples without bacteria, whereas the model trained solely on bacteria (unannotated) showed diminished *Recall* at high true negative rates of food samples because many bacterial microcolonies were not identified at this high confidence level. At a confidence level of 0.94, the model trained on both bacteria and food demonstrated 100% true negative rates for spinach, Cotija cheese, and chicken breast, while effectively classifying *E. coli*, *L. monocytogenes*, and *B. subtilis* with a *mPrecision* of 100% and a *mRecall* of 94.4%. This threshold was considered optimal because it eliminated false positives from food debris while maintaining a high level of recall, thereby balancing the practical need for specificity with the requirement for sensitive detection in food safety applications. Therefore, the deep convolutional neural network model, trained on both bacteria and food datasets, is robust for detecting and classifying bacteria in food, making it highly applicable for practical use in the food industry.

### Application of the deep learning model for detecting multispecies bacteria in food matrices

The performance of the deep learning model, trained on both bacteria and food debris, was further evaluated using representative food matrices spiked with GFP-producing *B. subtilis*. The fluorescence signal provides an independent validation of the target bacteria in the presence of food debris. These samples contained both bacterial microcolonies and food debris to simulate real-world detection scenarios. To generate ground truth annotations, *B. subtilis* microcolonies were identified by comparing phase-contrast and fluorescence images from the same field of view. Figure 6a shows phase-contrast images of *B. subtilis* microcolonies in different food matrices, including spinach, cheese, and chicken, while Fig. 6b represents the corresponding fluorescence images that confirm the presence of GFP-producing *B. subtilis*. As shown in Fig. 6c, the bacterial detection model effectively identified *B. subtilis* microcolonies in the presence of food debris (Fig. 6c). Across all food matrices, the model achieved a *mPrecision* of 94.6% and a *mRecall* of 92.5%. In total, 133 *B. subtilis* microcolonies were present across spinach, chicken, and cheese samples, of which 123 were correctly detected (true positives), while only 10 were missed (false negatives) and 7 food debris objects were misclassified as bacteria (false positives). Notably, at a confidence level of 0.94, the model also achieved a 0% false positive rate across all tested food matrices. These metrics highlight the robustness of the model in accurately classifying bacterial microcolonies while avoiding the misclassification of food debris as bacteria. This capability is critical for its practical application in the food industry, which enables rapid and reliable bacterial detection even in the presence of complex food matrices.

## Discussion

The interference of food debris presents a significant challenge for deep learning-based microbial detection in complex food matrices. Recently, deep learning models have been successfully developed to detect bacteria and yeast from microscopic images^5,8,9,12^, but few studies have investigated how food-derived interferences affect deep learning-based microbial detection performance. Ma et al. demonstrated that the AI-based *E. coli* detection model achieved 91.6% accuracy in detecting *E. coli* in romaine lettuce after 3 h of incubation^5^. However, the study did not evaluate the model’s performance on *E. coli*-negative samples, and thus, the false positive rate due to food debris (i.e., romaine lettuce) was not determined. Park et al. developed and validated a deep learning-based yeast detection model capable of classifying seven yeast species in tomato and tomato juice^12^. At a confidence level of 0.9, the model successfully excluded food debris from detection (*mPrecision* > 95.2% and *mRecall* > 93.9%). However, their yeast microcolonies were 50–100 μm, significantly larger than bacterial microcolonies investigated in this study (2–20 μm) (Fig. 2), which made the yeast detection model easily distinguish yeasts from food debris because yeast microcolonies were larger than food debris. The smaller size of bacterial microcolonies increases the likelihood of misclassification since they closely resemble various food debris objects (Fig. 2). In practical applications, food debris can cause false positives due to misclassification as bacteria, which reduces detection accuracy. To improve the robustness of deep learning-based bacterial detection in complex food matrices, more refined model development strategies must account for the interference introduced by food debris.

This study illustrates an approach to minimize the interference of food debris for the detection of target bacteria in food samples, which improves the practical application of a deep learning-based rapid bacterial detection model. When the deep neural network model was trained solely on bacterial microcolony images, food debris were misclassified as bacteria (Fig. 4a). Although this model training approach allowed the model to effectively distinguish between selected bacterial species (*L. monocytogenes*, *E. coli*, and *B. subtilis*) based on their unique morphological features (Fig. 3a), it lacked the necessary reference information to differentiate bacterial microcolonies from visually similar food debris. Without food debris images in the training dataset, the model applied its bacterial classification criteria too broadly, which led to the misclassification of non-bacterial particles with similar morphologies. This limitation highlights the need to include all relevant classes in the training dataset since incomplete training data can lead to misclassification of untrained objects^25^. To overcome this limitation, the model was refined by including images of three representative food debris (chicken breast, spinach, and Cotija cheese) in the training process. This adjustment improved the discrimination of the classification model between bacterial microcolonies and morphologically similar food debris. As a result, the model’s performance significantly improved **(**Fig. 4b), which could enhance its applicability in real-world scenarios where microscopic food debris is commonly present.

**Fig. 7:**
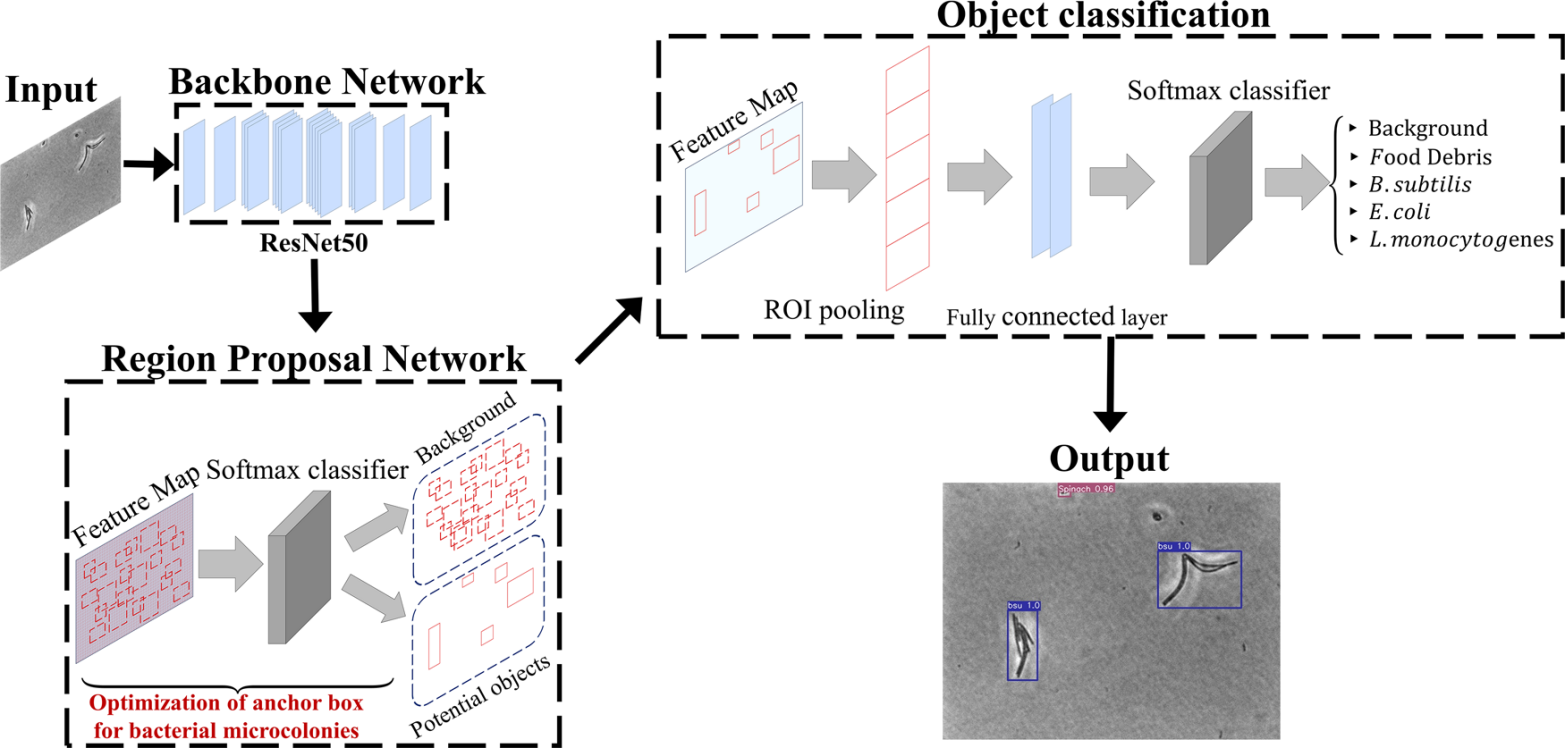
Architecture of the bacterial microcolony detection model using white light microscopy images as input, followed by a convolutional backbone network, a regional proposal network, and object detection and classification.

In addition to Faster R-CNN, a YOLOv7-based model was also trained on the same image dataset to evaluate whether the proposed bacterial detection approach based on microcolony imaging is robust across different model architectures. A description of the YOLOv7-based model and its performance on bacterial classification is provided in the Supplementary Information (Supplementary Note 1). As shown in Supplementary Fig. 1, the overall bacterial detection accuracy of YOLOv7 model without training on debris varied from 0.482 (at a confidence level of 0.9) to 0.978 (at a confidence level of 0.7). Trained on bacterial and food debris images, the YOLOv7 model could achieve a 100% true negative rate of unseen data (Supplementary Figs. 2 and 3). At the confidence thresholds yielding the best overall classification performance, Faster R-CNN achieved an *mPrecision* of 100% and an *mRecall* of 94.4%, whereas YOLOv7 achieved an *mPrecision* of 97.0% and an *mRecall* of 92.3%. In this study, Faster R-CNN consistently outperformed YOLOv7 in terms of classification accuracy and false positive reduction. Several studies have compared the performance of Faster R-CNN and YOLOv7 architectures for deep learning-based object detection. While YOLOv7 has demonstrated competitive or superior performance in detecting common real-world objects such as pedestrians, cars, and bicycles^26–28^, Faster R-CNN has shown advantages in detecting small or fine-grained targets. For instance, Wu et al. reported that Faster R-CNN outperformed YOLOv7 in identifying small and structurally variable catenary components^29^. Similarly, Cui et al. found that Faster R-CNN more effectively classified the growth stages of soybean seedlings in UAV imagery, where the targets were relatively small compared to the background^30^. These findings are consistent with our results, where Faster R-CNN achieved higher classification accuracy and reduced false positives in detecting and classifying small bacterial microcolonies. This improved performance is likely due to architectural differences: Faster R-CNN incorporates a RPN to generate candidate object regions, enabling more precise localization of small or partially occluded objects (Fig. 7). In contrast, YOLOv7 performs direct bounding box regression on feature maps using predefined anchors, which can reduce its accuracy for small or irregularly shaped microcolonies embedded in noisy backgrounds such as food debris^31^. These differences highlight the importance of selecting a suitable model architecture based on the size, contrast, and complexity of the target features. Our findings further support the use of region proposal-based models for microbial image analysis tasks where subtle morphological cues and high noise levels often coexist.

Evaluation of new microbial detection methods using inoculated bacteria in food matrices is essential, as the complexity of food matrices may significantly impact detection accuracy^11,32,33^. However, validating image-based microbial detection models in food matrices is particularly challenging due to the absence of independent ground truth annotations that can reliably distinguish microorganisms from visually similar food debris in images. In this study, GFP-producing *B. subtilis* was successfully employed to overcome this limitation, enabling precise discrimination of bacteria from food debris. By leveraging the fluorescence signal of GFP-producing *B. subtilis*, accurate ground truth annotations were created, which facilitate effective validation of the bacterial detection model in the presence of food debris (Fig. 6). This approach demonstrated the practical applicability of the bacterial detection model for real-world food matrices. The results of this study suggest that the novel method of using fluorescence-producing bacteria can serve as a valuable tool for validating deep learning-based image analysis in bacterial detection. Furthermore, the bacterial detection model developed in this study achieved rapid detection of foodborne bacteria within 3 h, which meets the requirement of rapid turnaround analysis for the food industry. Nevertheless, the current framework still faces a critical limitation, as it does not provide robust species-level identification across diverse foodborne bacteria and natural food microflora. Distinguishing pathogenic species from non-pathogenic background microflora is essential for adoption in the food industry, as misclassification would reduce the practical utility of rapid screening tools. Future research will therefore need to focus on (i) expanding datasets to include naturally occurring food microflora together with target pathogens and various food types, (ii) developing hierarchical AI models that first discriminate bacteria from food debris and then refine species-level classification, and (iii) integrating complementary sensing modalities such as fluorescence, hyperspectral imaging, or molecular assays to provide orthogonal features for discrimination. Broader validation under these diverse scenarios will be essential to achieve robust, species-level classification. Addressing this challenge will enable the consistent and reliable deployment of AI-based bacterial detection models in complex food systems.

## Methods

### Bacterial strains, culture preparation, and food sample preparation

To develop an AI-based bacterial detection and classification method, three bacterial strains were selected in this study: *E. coli* (LJH 1612) isolated from irrigation water^5^, *B. subtilis* (ATCC 23857) isolated from soil^34^, and *L. monocytogenes* (LIS 0110) isolated from a cantaloupe outbreak^35^. Additionally, a GFP-producing *B. subtilis* strain (YC161)^36^ was used to validate the AI-based bacterial detection and classification method. These four strains were preserved at −80 °C in tryptic soy broth (TSB) containing 20% (v/v) glycerol for the cryopreservation of bacterial cells. For culture preparation, the glycerol stocks were streaked onto tryptic soy agar (TSA; 1.5% agarose, w/v) and incubated overnight at 37 °C. A single colony was then transferred from the TSA plate to tryptic soy broth, with shaking at 175 rpm at 37 °C for 24 h in the case of *E. coli* and 48 h for both *B. subtilis* and *L. monocytogenes*. The prepared bacterial cultures were diluted in 1x phosphate-buffered saline (PBS, pH 7.4) to obtain the desired concentrations for further experimentation.

Three different types of food samples were tested, including raw chicken breast, spinach, and Cotija cheese. Raw chicken breast, baby spinach leaves, and Cotija cheese were purchased from a local grocery store and used within 2 h after purchasing. To simulate the workflow of food sample analysis, food samples were homogenized in 1x PBS using a stomacher (Seward Stomacher 400 Circulator, Hamilton, NJ). Briefly, food samples (10 g of spinach leaves and 25 g of chicken breast or Cotija cheese) were transferred into a sterile sampling bag containing 1x PBS for two-fold dilution (20 mL for spinach samples and 50 mL for chicken and cheese samples). The sampling bags were sealed and blended using the stomacher at 300 rpm for 90 s. After homogenization, the supernatant of food samples was transferred to 50-mL Falcon tubes for immediate analysis or stored at 4 °C.

### Images of bacterial microcolony and food debris

To discriminate various bacteria in the presence of food debris using optical imaging and deep learning algorithms, individual bacterial cultures and food debris were prepared separately to create an image dataset for training the deep learning models. The procedure was performed according to our previous protocol^5^. For bacterial image data, 10 μL of bacterial culture was deposited onto 60-mm soft TSA plates (0.7% agarose, wt/vol), followed by incubation at 37 °C for 3 h to allow microcolony formation. For food image data, 10 μL of an aliquot of food samples was deposited on the soft TSA plates, followed by air drying at 37 °C for 10 min. The absence of a 3-h incubation period for food samples at 37 °C will limit the formation of microcolonies from the natural microflora in foods due to a lack of an enrichment step, making it easier to label food debris for training deep learning models. Since the model performs object-level detection rather than image-level classification, each bacterial microcolony is treated as an individual instance. Therefore, the initial concentration of bacterial cells does not affect detection performance but only influences the number of microcolonies formed per plate and thus the number of objects available for annotation and training. Afterwards, bacterial microcolonies and food debris on agar were observed using a conventional phase-contrast microscope (IX71, Olympus, Center Valley, PA) with a 60×/0.7 Ph2 Air objective (Olympus LUCPlanFL N) under white light illumination (TH4-100, Olympus). Microscopic images were acquired in PNG format (672 × 512 pixels with a pixel size of 107.5 nm) using an ORCA-ER digital camera (Hamamatsu, Japan). Microscopic images were obtained from three independent experiments conducted on separate days. For each bacterial strain, 105 images were collected from three plates per day (total of 315 images per strain). For each food sample, 70 images were collected from three plates per day (total of 210 images per sample). The images were converted to JPG format for further analysis, and min-max normalization was applied during preprocessing to reduce variations in image brightness and contrast. To provide ground truth annotations, bounding boxes were created around bacterial microcolonies and food debris using an open-source annotation tool (Computer Vision Annotation Tool, Intel, Santa Clara, CA). The annotation files for each image were then extracted in XML format for subsequent processing.

### Deep learning model architecture and training

To detect and classify *E. coli*, *L. monocytogenes*, and *B. subtilis* in the presence of food debris, the bacterial detection model was developed based on the architecture of Faster R-CNN^24^, pre-trained on MS COCO train 2017^37^. The architecture of bacterial detection models consists of the following components (Fig. 7):

Backbone network: The ResNet50 backbone network serves as a feature extractor, analyzing input images to capture details and patterns at multiple scales. It produces feature maps that provide a detailed representation of the morphological characteristics.

Region Proposal Network (RPN): The feature maps from ResNet50 are then passed to the RPN, which uses a sliding window mechanism to generate anchor boxes of various scales and aspect ratios across the feature map. For each anchor box, the RPN performs binary classification via softmax function to determine the presence of an object and applies bounding box regression to refine its coordinates. In this study, anchor box parameters were optimized to match the small size and aspect ratios of bacterial microcolonies.

Region of Interest (ROI) pooling: The identified ROIs are processed through ROI pooling, which extracts the corresponding features from the feature maps and resize them to a fixed spatial size. This standardization ensures uniform input dimensions for the subsequent fully connected layers, which allows consistent processing and preserving spatial information relevant to object classification and localization.

Output for class discrimination: The features from each ROI are further processed through fully connected layers followed by a softmax classifier, which assigns the detected object to one of the bacterial species, food debris, or background. In parallel bounding box regression layers adjust the coordinates to refine the localization of each detected object with improved precision.

By training on annotated bacterial microcolonies, the model was configured to generate bounding box coordinates and class predictions for bacterial microcolonies based on their morphological features in the presence of food debris^24^.

For model training, the preprocessed images were divided into three subsets: 60% for training, 10% for validation, and 30% for testing. Two different training strategies were employed to evaluate the impact of including food debris images on the model’s performance. In the first strategy, the model was trained exclusively on images of bacterial microcolonies (*E. coli*, *L. monocytogenes*, and *B. subtilis*), without incorporating any food debris images in the training and validation datasets. In the second strategy, the model was trained on a combined dataset containing both bacterial microcolonies and food debris images (chicken breast, spinach, and Cotija cheese). To enhance training efficiency, data augmentation techniques such as rotation, flipping, and brightness adjustment were employed. The model was trained using a stochastic gradient descent optimizer with a batch size of 4, a learning rate of 0.00002, weight decay of 0.0005, and a momentum of 0.9. The validation dataset was used to optimize the model by selecting the best hyperparameter settings during the training process. The training was conducted on a high-performance computing system, utilizing a Tesla A100 40GB GPU (NVIDIA, Santa Clara, CA).

### Evaluation of model performance

After training, the performance of the bacterial detection model was evaluated using a separate test dataset and the following metrics:

1 $${Precision}=\,\frac{{TP}}{{TP}+{FP}}$$

2 $${Recall}=\frac{{TP}}{{TP}+{FN}}$$

3 $${mPrecision}=\frac{1}{n}\mathop{\sum }\limits_{i=1}^{i=n}{P}_{i}$$

4 $${mRecall}=\frac{1}{n}\mathop{\sum }\limits_{i=1}^{i=n}{R}_{i}$$

5 $$\mathrm{True}\mathrm{negative}\mathrm{rate}=\left(1-\frac{{n}_{f,b}}{{n}_{f,T}}\right)\times 100$$

where *TP*, *FP*, and *FN* are true positive, false positive, and false negative, respectively. *P*_*i*_ and *R*_*i*_ are the precision and recall of class *i*. *mPrecision* and *mRecall* are the mean of precisions and recalls across all classes, respectively. ${n}_{f,T}$ is the total number of food debris images and ${n}_{f,b}$ is the number of food debris images that were mistakenly identified as bacteria.

### Application of the deep learning model for detecting multispecies bacteria in the presence of food matrices

The performance of the bacterial detection model was further evaluated in real-world scenarios. To simulate the real-world samples, 10 μL of the GFP-producing *B. subtilis* culture (YC161) was inoculated on food samples (10 g of spinach leaves and 25 g of chicken breast or Cotija cheese). The inoculated samples were then placed in Whirl-Pak homogenization bags with a 330 μm mesh filter (Pleasant Prairie, WI, USA) containing PBS (20 mL for the spinach sample and 50 mL for the chicken and cheese samples). Each bag was homogenized in the stomacher at 300 rpm for 90 s. From the homogenized samples, 10 μL of an aliquot was spread-plated onto 60-mm soft TSA plates and incubated for 3 h at 37 °C to allow microcolony formation. For the validation experiments, images were obtained from two independent experiments conducted on different days. For each sample, 10 microscopic images were captured from three plates, resulting in a total of 20 images per food matrix and a total of 60 images across the three matrices. Ground-truth annotations for *B. subtilis* microcolonies were created as described in section “Images of bacterial microcolony and food debris”. To annotate the *B. subtilis* microcolonies in the presence of food debris, bacterial microcolonies of GFP*-*producing *B. subtilis* were identified by comparing phase-contrast images with fluorescence images using a fluorescein isothiocyanate (FITC, excitation at 480 nm and emission at 515 nm) filter in the same field of view. To reduce the influence of background noise and enhance the GFP signal, a background subtraction-based image preprocessing pipeline was implemented in Python using NumPy and OpenCV. A per-pixel mean background image was generated from the entire fluorescence image stack and subtracted from each individual image after normalization. This approach effectively suppressed consistent non-specific background fluorescence and improved the detectability of a relatively weak GFP signal from microcolonies on agar plates. The resulting enhanced fluorescence images were then used to generate ground truth annotations of *B. subtilis* microcolonies.

## Supplementary information

Supplementary Information

## Data Availability

The data that support the findings of this study are available from the corresponding author upon reasonable request.

## Code availability

The code supporting this study will be made available at https://github.com/AI-Institute-Food-Systems.
