# Supplementary material for: Deep learning enabled rapid detection of live bacteria in the presence of food debris
Source: NPJ Sci Food. 2025 Nov 21;9:274. doi: 10.1038/s41538-025-00636-z (PMC12728209; doi:10.1038/s41538-025-00636-z)
Supplement: Supplementary file 1 — Supplementary Information [file 41538_2025_636_MOESM1_ESM.pdf]

Supplementary Information

Deep learning enabled rapid detection of live bacteria in the presence of food debris

Hyeon Woo Park[a, b, ‡], *Zhengao Li*[c, ‡], *Luyao Ma*[d, e, *], *and Nitin Nitin*[b, f, *]

AUTHOR ADDRESS

[a] Department of Food & Biotechnology, Korea University, Sejong 30019, Republic of Korea

[b] Department of Food Science & Technology, University of California-Davis, Davis, CA 95616, USA

[c] Department of Computer Science, Florida State University, Tallahassee, FL 32306, USA

[d] Department of Food Science & Technology, Oregon State University, Corvallis, OR 97331, USA

[e] Department of Biological & Ecological Engineering, Oregon State University, Corvallis, OR 97331, USA

[f] Department of Biological & Agricultural Engineering, University of California-Davis, Davis, CA 95616, USA

‡ Co-first author with equal contributions.

[*] Co-corresponding author: luyao.ma@oregonstate.edu (L. Ma); nnitin@ucdavis.edu (N. Nitin).

Supplementary Note 1

The architecture of YOLOv7 model consists of the following components.

1) Backbone: A feature extractor used to capture features from the input image. YOLOv7 uses a modified version of CSPDarknet53 as its backbone, which balances accuracy and speed by incorporating cross-stage partial network (CSP).

2) Neck: This component aggregates features from different levels of the backbone. YOLOv7 utilizes a feature pyramid network (FPN) to help the model detect objects at different scales.

3) Head: The detection head in YOLOv7 is where the final predictions are made, including object classification and bounding box regression. The head processes the aggregated features from the neck and predicts bounding boxes along with confidence scores and class labels.

For YOLOv7 training, the preprocessed images were split into three subsets: 60% for trai ning, 10% for validation, and 30% for testing. The training utilized the COCO pre-trained weights to enhance the model's performance on our specific dataset. Mosaic augmenta tion was applied with a probability of 50% to further improve generalization. The model was trained for 300 epochs. The learning rate was scheduled to decay from an initial rate of 0.01 t o a minimum of 0.0001 using a cosine annealing strategy. Training was conducted on a high-performance computing system equipped with an NVIDIA RTX 4090 24GB GPU.

Figures

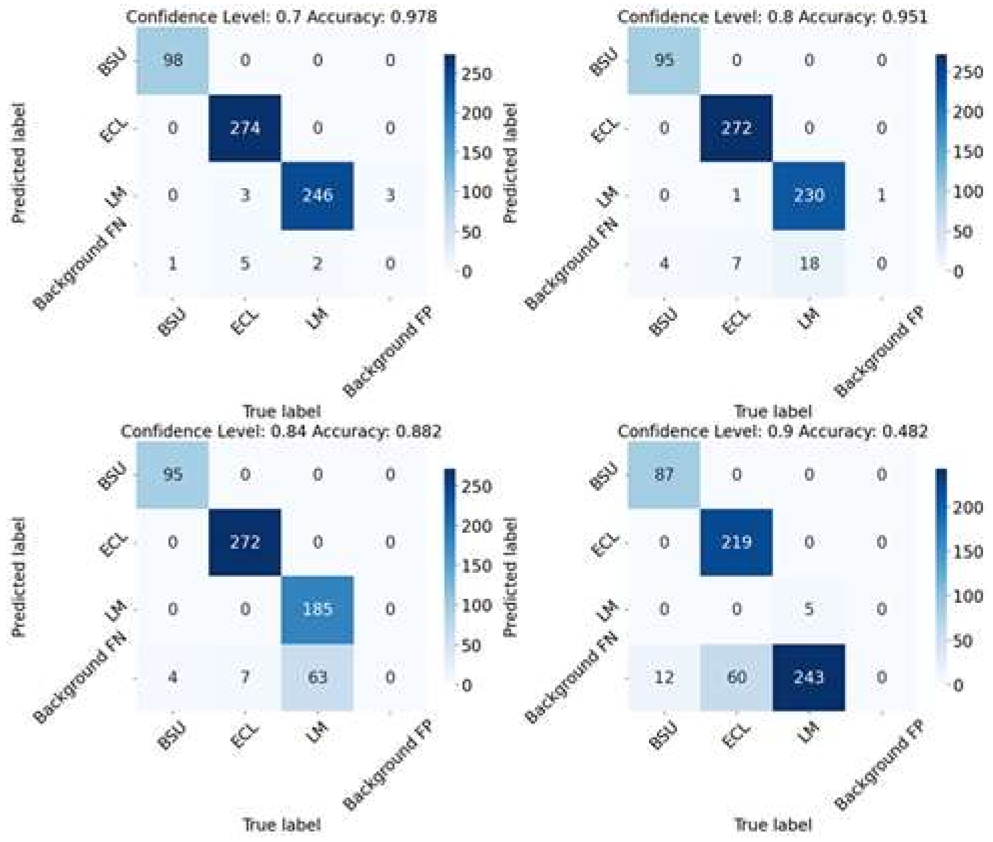

Supplementary Figure 1. Confusion matrix at different confidence levels for bacterial classification using the YOLOv7 model trained on *Listeria monocytogenes*, *Escherichia coli*, and *Bacillus subtilis*.

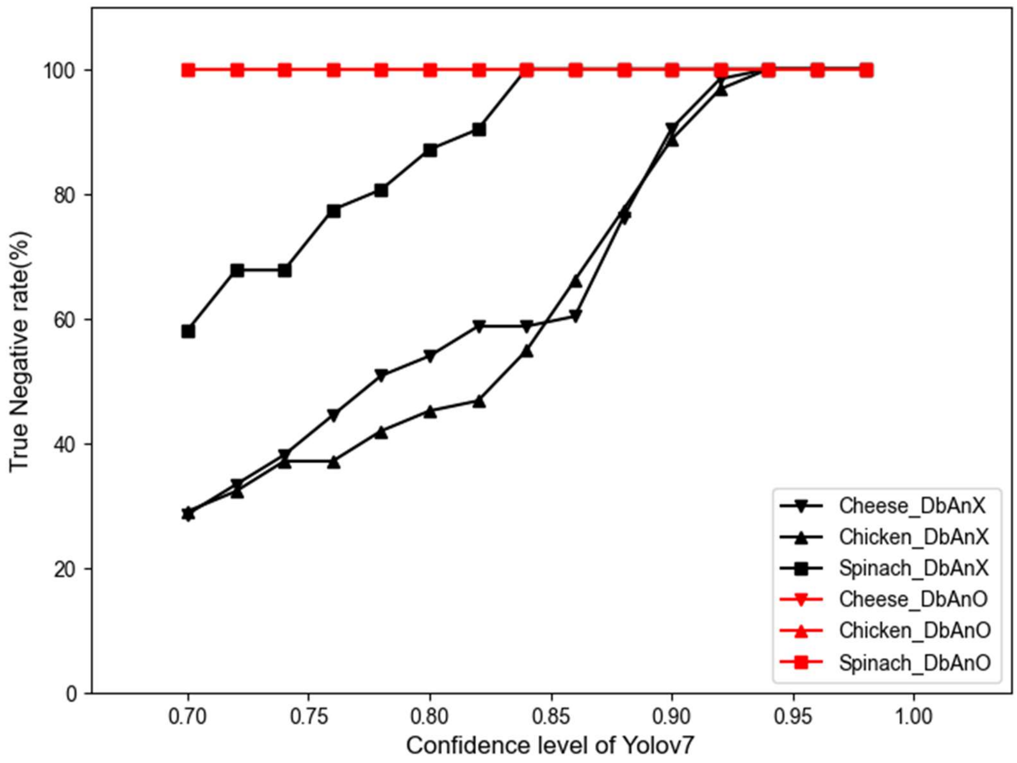

Supplementary Figure 2. True negative rates of food debris images at different confidence levels for the YOLOv7 models trained on bacteria (DbAnX) and bacteria with food (DbAnO).

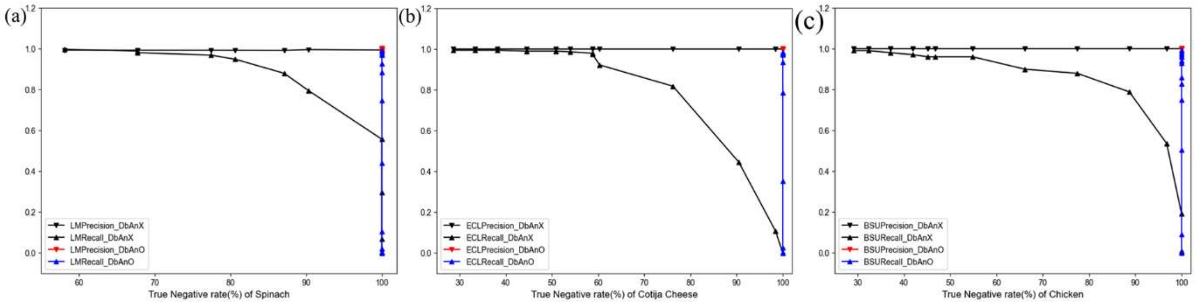

Supplementary Figure 3. Precision and recall curves of bacteria in relation to the true negative rate of corresponding food debris: (a) *Listeria monocytogenes* and spinach, (b) *Escherichia coli* and Cotija cheese, and (c) *Bacillus subtilis* and chicken breast. “DbAnX” and “DbAnO” refer to the YOLOv7 models trained on bacteria only and bacteria with food, respectively.
